# Elevated *Toxoplasma gondii* Infection Rates for Retinas from Eye Banks, Southern Brazil

**DOI:** 10.3201/eid2204.141819

**Published:** 2016-04

**Authors:** Alessandra G. Commodaro, Melissa Chiasson, Natarajan Sundar, Luiz Vicente Rizzo, Rubens Belfort, Michael E. Grigg

**Affiliations:** National Institutes of Health, Bethesda, Maryland, USA (A.G. Commodaro, M. Chiasson, N. Sundar, M.E. Grigg);; Federal University of São Paulo, São Paulo, Brazil (A.G. Commodaro, R. Belfort);; Hospital Albert Einstein, São Paulo (L.V. Rizzo)

**Keywords:** ocular toxoplasmosis, serotype, *Toxoplasma gondii*, genotype, retina, parasites

## Abstract

We found significantly higher incidence of *Toxoplasma gondii* DNA in eye bank specimens from Joinville in southern Brazil (13/15, 87%) than in São Paulo (3/42, 7%; p = 2.1 × 10E–8). PCR DNA sequence analysis was more sensitive at locus *NTS2* than at locus *B1*; a high frequency of mixed co-infections was detected.

*Toxoplasma gondii* is a protozoan parasite that infects nearly one third of the world’s human population. Infection is typically asymptomatic, but life-threatening disease may develop in immunocompromised patients and when the infection is acquired congenitally (*1*). When parasites migrate to the eye, ocular toxoplasmosis (OT) can occur; this potentially blinding disease causes a high incidence of uveitis worldwide (*1*). In the United States, ≈2% of *Toxoplasma* infections progress to OT, whereas in southern Brazil, the proportion can be >18% (*1*). Factors leading to development of OT are poorly understood. In most OT studies, the study population is symptomatic persons. Little is known about OT infection status among a random population. We investigated the incidence of OT in a random population in Brazil and determined the sensitivity of 3 PCR diagnostic markers used for detecting parasite DNA and genotype. 

## The Study

**Figure 1:**
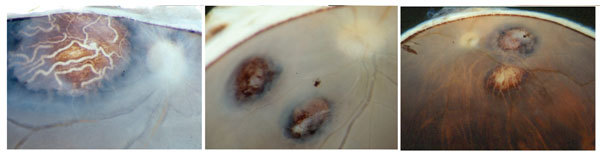
Representative retinochoroidal lesions depicted for 3 of 18 archived eyes examined by optical microscopy for evidence of *Toxoplasma gondii* infection; 270 single eyes were examined, each from a different donor, obtained during 1985 and 2009 in São Paulo, São Paulo State, Brazil. Original magnification (left to right): ×20,

In southern Brazil, incidence of OT ranges from 9.8% to 22% (*2*–*4*). In 1985, as part of a study to determine prevalence of toxoplasmic retinochoroidal lesions among a random population, 270 archived eyes submitted to the São Paulo Eye Bank consecutively were examined for macroscopic chorioretinal lesions or histologic evidence of retinochoroiditis (R. Belfort, Jr., unpub. data). Each was from a different person (no bilateral specimens were received). Eighteen (6.7%) eyes had chorioretinitis and 9 (3.3%) possessed cicatrial lesions; total prevalence was 10% (27/270). Of these 27 eyes, 8 (3.0%) had mononuclear cells, and 1 (0.4%) had acute, inflammatory infiltrate consistent with OT (Figure 1). These eyes were no longer available for molecular testing.

**Figure 2:**
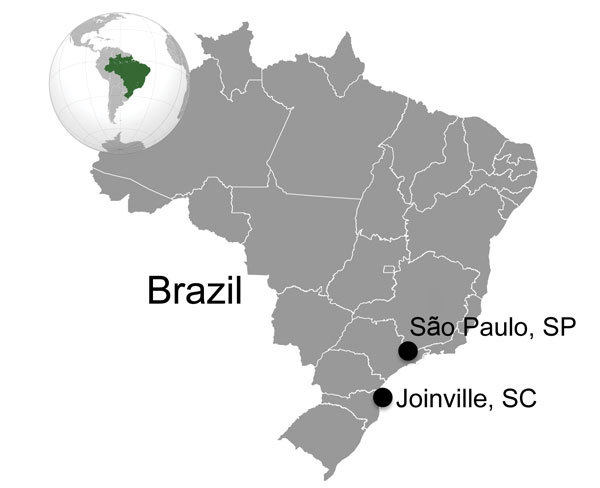
Cities where retinochoroidal scars consistent with *Toxoplasma gondii* infection were found on eyes donated to eye banks in Brazil during 1985 and 2009: São Paulo, São Paulo State, and its relative position in southern Brazil to Joinville, Santa Catarina State; 522 km separates the 2 cities.

To determine updated infection prevalence rates, we obtained 114 eyes collected during 2009 from eye banks in 2 disease-endemic regions of Brazil with different OT incidence rates, the south (Joinville, n = 15) and southeast (São Paulo, n = 42) (Figure 2). The eyes were collected consecutively from deceased persons who donated to eye banks, 15 in Joinville and 42 in São Paulo. All eyes were from patients who died of natural causes or in an accident and who had no history or symptoms of ophthalmologic disease. The average age of donors was 54 years (range 20–76 years); exclusion criteria included a history of lymphoma, leukemia, leptospirosis, hepatitis B or C, HIV, rabies, or bacteria associated with endocarditis. Matched serum samples for each eye were not available. 

We extracted DNA from eyes to ascertain *T. gondii* prevalence and *Toxoplasma* genotype. Eyes were not examined macroscopically. Retinal DNA was extracted by using a QIAamp DNA Blood Midi Kit (QIAGEN, Valencia, CA, USA). Multilocus PCR DNA sequencing was performed by using B1, NTS2, and GRA7 loci, as described (*5*,*6*). Positive (type I RH DNA) and negative (water only) controls were run alongside each PCR. PCR products were treated with ExoSAP-IT (USB Corp., Cleveland, OH, USA) before DNA sequencing (RML Genomics, Hamilton, MT, USA).

Specimens from 3 (7.1%) of 42 donors from São Paulo tested positive at NTS2 (Technical Appendix Table), similar to the incidence rate determined among the eyes examined macroscopically in 1985 and consistent with previously published work (*7*). Our results show that no significant change in relative frequency of *Toxoplasma* infection occurred in eyes donated in São Paulo over a 25-year period. DNA sequence analysis of the 3 positive samples identified an archetypal allele shared by types II and III strains at NTS2. Only 1 NTS2-positive eye (no. 37) was positive at B1 (sensitivity 33% relative to NTS2; B1 is a standard diagnostic marker [*5*]). It possessed a unique allele (Technical Appendix Table). No samples were PCR positive at the GRA7 marker.

Of the 15 donors from Joinville, 13 (87%) tested positive for *Toxoplasma* DNA at NTS2. Six samples possessed type I alleles, 1 had a unique allele sharing phylogenetic ancestry with type I, 2 had drifted type II or type III alleles, and 4 possessed a mixture of 2 allelic types, indicative of mixed strain co-infection. The detection of 5, 3, and 3 distinct and mostly novel alleles at NTS2, B1, and GRA7, respectively, argue against the possibility that the PCR results were due to a false-negative amplification. A high incidence of mixed infection has been observed previously in Australian marsupials (*8*), but it is considered rare in human infection (*9*). In contrast, only 6 samples were positive at B1 (sensitivity 46% relative to NTS2); 1 sample had a type I allele, 4 had a unique allele, and 1 (no. 5) had a mixture of 2 different alleles. Three samples were positive at GRA7 (sensitivity 23% relative to NTS2); a type I allele, and 2 alleles sharing phylogenetic ancestry with type I and type III, respectively (Technical Appendix Table).

Southern Brazil experiences different incidence rates and severity of OT, although overall toxoplasmosis prevalence rates are similar. Studies in metropolitan São Paulo identified *Toxoplasma* antibodies in samples from pregnant patients that ranged from 59% to 65% across 2 decades, but no data on frequency of OT was reported (*10*,*11*). One 2004 study showed that ≈10% of the metropolitan Sao Paulo population has OT (*7*). Seroprevalence in São Jose do Rio Preto, another city in São Paulo State, is 74.5%, and the incidence of OT in 2009–2010 was 20.3% (*2*). Seropositivity in Erechim, a southern city in Rio Grande do Sul, is ≈88%; OT occurs frequently (18%) and is severe (*12*). 

## Conclusions

Our results reveal differences in the ability of parasites to infect eyes collected from the general population in different geographic regions of Brazil. The difference in eye infection prevalence rates suggests that, although most of the São Paulo population is seropositive, only 7% of the population harbor *Toxoplasma* parasites capable of migrating to the eye. However, 87% of eyes from Joinville tested positive for parasite DNA (a 1:1 correlation with seroprevalence), which may indicate that no apparent barrier to parasite entry into the eye exists in this geographic region. Although not all patients bearing parasite infections in their eyes develop OT, similar to the finding that only 30%–50% of AIDS patients experience recrudescent disease (*13*), our results may explain the increased incidence of OT in southern Brazil and help clarify possible factors controlling the disease.

Our findings also show that the 110 gene-copy NTS2 marker proved more sensitive than B1, a marker traditionally used to detect infection (*5*). In addition, NTS2 polymorphisms enabled mixed strain co-infections to be detected in the eyes of donor no. 5. Multilocus typing established that atypical strains common to Brazil caused most eye infections and identified different *Toxoplasma* genotypes circulating between the 2 locations; e.g., strains bearing alleles that share phylogenetic ancestry with type I were only identified in Joinville. Previous studies suggested a link between parasite genotype and development of OT (*14*). Our work using serologic typing has established that the parasite genotype is associated with more serious congenital and ocular disease among patients in the United States and Europe (*6*,*15*). Ultimately, to determine whether increased eye infection rates in Joinville is dependent on parasite genotype, or an associated host genetic risk factor, prospective sampling should be done on eyes donated to eye banks. The serostatus of the donor, whether retinal scars are present, and the geographic origin of the donor can be recorded to determine factors associated with parasite infection and whether such donated eyes should be precluded from transplantation.

Technical Appendix Polymorphisms in the NTS2, B1, and GRA7 genes by direct PCR on *Toxoplasma gondii*–infected eyes identified in eye banks from Joinville and São Paulo City, Brazil. 
