# Supplementary material for: Elevated Toxoplasma gondii Infection Rates for Retinas from Eye Banks, Southern Brazil
Source: Emerg Infect Dis. 2016 Apr;22(4):691–3. doi: 10.3201/eid2204.141819 (PMC4806975; doi:10.3201/eid2204.141819)
Supplement: Technical Appendix — Polymorphisms in the NTS2, B1, and GRA7 genes by direct PCR on Toxoplasma gondii–infected eyes identified in eye banks from Joinville and São Paulo City, Brazil. [file 14-1819-Techapp-s1.pdf]

DOI: http://dx.doi.org/10.3201/eid2204.141819

Elevated *Toxoplasma gondii* Infection Rates for Retinas from Eye Banks, Southern Brazil

Technical Appendix

Technical Appendix Table. Polymorphisms in the NTS2, B1, and GRA7 genes by direct PCR on *Toxoplasma gondii*–infected eyes identified in eye banks from Joinville and Sao Paulo City, Brazil

	NTS2								B1					GRA7																	
	723	761	765	843	856	912	1009	Allele	225	366	378	504	Allele	396	402	446	502	505	523	533	540	550	568	577	605	619	625	629	683	690	Allele
Joinville																															
Consensus	T	G	T	A	G	T	A		C	t/c	G	g/c		A	T	A	A	C	C	C	C	A	A	T	C	A	C	C	T	A	
Type I	C	T	A	G	A	.	.	I	.	T	.	G	I	G	.	.	C	G	G	G	.	.	G	.	.	.	.	.	.	.	I
Type II	.	.	.	.	.	.	.	II	.	.	.	.	II	.	.	.	.	.	.	.	A	.	.	.	.	.	.	.	.	.	II
Type III	.	.	.	.	.	.	.	III	.	.	.	.	III	.	.	.	.	.	.	.	.	T	.	A	T	C	A	A	C	C	II
1-LE	C	T	A	G	A	.	.	I																							
2-RE	C	T	A	G	A	.	.	I																							
3-RE	C	T	A	G	A	.	g/a	u1																							
4-RE	t/c	g/t	a/t	g/a	g/a	.	.	mixed	.	T	.	C	u1	G	A	.	C	G	G	G	.	.	G	.	.	.	.	.	.	.	u1
5-LE	t/c	g/t	a/t	g/a	g/a	.	.	mixed	.	T	.	C	u1																		
5-RE	.	.	.	.	.	.	.	II/III	T	T	a/g	G	u2																		
6-RE	C	T	A	G	A	.	.	I						x	x	G	.	.	.	.	.	T	.	A	T	C	A	A	C	.	u2
7-RE	C	T	A	G	A	.	.	I																							
8-LE														G	.	.	C	G	G	G	.	.	G	.	.	.	.	.	.	.	I
9-LE	C	T	A	G	A	.	.	I	.	T	.	G	I																		
10-LE	.	.	.	.	T	C	.	u2	.	T	.	C	u1																		
11-RE	t/c	g/t	a/t	g/a	g/a	.	.	mixed	.	T	.	C	u1																		
12-RE	.	.	.	.	T	.	.	u3																							
14-LE	C	T	A	G	A	.	.	I																							
14-RE	C	T	A	G	A	.	.	I																							
15-RE	t/c	g/t	a/t	g/a	g/a	.	.	mixed																							
São Paulo																															
34	.	.	.	.	.	.	.	II/III																							
37	.	.	.	.	.	.	.	II/III	.	T	.	C	u1																		
49	.	.	.	.	.	.	.	II/III																							

Reference AF158092-RH AF179871-RH DQ459443-RH
